# Evaluating the Effectiveness of Neoadjuvant Therapy in Her2-Positive Invasive Breast Cancer: A Comprehensive Analysis of 167 Cases in Romania

**DOI:** 10.3390/cancers17142312

**Published:** 2025-07-11

**Authors:** Bogdan Pop, Carmen Popa, Nicoleta Zenovia Antone, Patriciu-Andrei Achimaș-Cadariu, Ioan-Cătălin Vlad, Gabriela Morar-Bolba, Daniela Laura Martin, Carmen Lisencu, Călin Căinap, Roxana Pintican, Annamaria Fulop, Cosmin Ioan Lisencu, Codruț Cosmin Nistor-Ciurba, Maximilian Vlad Muntean, Cătană Andreea, Bogdan Fetica

**Affiliations:** 1 Department of Morpho-Functional Sciences, “Iuliu Hatieganu” University of Medicine and Pharmacy, 400012 Cluj-Napoca, Romania; 2 Department of Pathology, “Prof. Dr. Ion Chiricuta” Institute of Oncology, 400015 Cluj-Napoca, Romania; 3 Radiotherapy Department, “Prof. Dr. Ion Chiricuta” Institute of Oncology, 400015 Cluj-Napoca, Romania; 4 Oncology Department, “Iuliu Hatieganu” University of Medicine and Pharmacy, 400012 Cluj-Napoca, Romania; calincainap2015@gmail.com (C.C.);; 5 Oncological Surgery Department, “Prof. Dr. Ion Chiricuta” Institute of Oncology, 400015 Cluj-Napoca, Romania; 6 Medical Oncology, “Prof. Dr. Ion Chiricuta” Institute of Oncology, 400015 Cluj-Napoca, Romania; 7 Department of Radiology, “Iuliu Hatieganu” University of Medicine and Pharmacy, 400012 Cluj-Napoca, Romania; 8 Department of Radiology, “Prof. Dr. Ion Chiricuta” Institute of Oncology, 400015 Cluj-Napoca, Romania; 9 Department of Molecular Sciences, “Iuliu Hatieganu” University of Medicine and Pharmacy, 400012 Cluj-Napoca, Romania; 10 Department of Genetic Explorations, “Prof. Dr. Ion Chiricuta” Institute of Oncology, 400015 Cluj-Napoca, Romania

**Keywords:** breast cancer, pathological complete response, Her2

## Abstract

Pathological complete response (pCR) following neoadjuvant therapy for IBC has shown a strong correlation with event-free survival and overall survival. Over the past three decades, the five-year net survival rate for breast cancer has generally increased; however, several Eastern European countries exhibit lower survival rates. Data from Romania, specifically regarding Her2-positive breast cancer response to therapy, are notably limited. **Background/Objectives**: The aim of our study was to evaluate the response to NAT using chemotherapy and Her2-targeted therapy in a cohort of 167 patients diagnosed with invasive breast cancer in our institution. **Methods**: We retrospectively analyzed 167 consecutive cases diagnosed with IBC in our institution between January 2020 and September 2024 with Stages II and III Her2-positive IBC. The overall pCR rates and several factors cited in the literature as predictors of pCR were analyzed. **Results**: Overall, the pCR rate was 50.29%, with higher values in 3+ cases (62.28%) compared to 2+ cases/ISH amplified (24.53%). Higher pCR rates were observed in hormone-negative cases, Stage II cases, estrogen receptor-negative cases, and high Ki-67 values. Patient age, ISH group, Her2 copy number, Her2:CEP17 ratio, and clinical lymph node involvement did not seem to influence pCR rates in our study. **Conclusions**: The data presented in our study represent, to the best of our knowledge, the largest cohort of patients diagnosed with Her2-positive IBC from Romania. The presented results and the pCR predictive factors were comparable to those cited in other studies on Her2-positive IBC cases.

## Simple Summary

This study investigated treatment efficacy for 167 patients with invasive breast cancer. The treatment included chemotherapy and a targeted therapy for Her2-positive cancer. The overall success rate was 50.29%, meaning that in about half of patients the histopathological examination of resection specimens revealed a complete absence of invasive tumor cells in the breast tumor and the absence of tumor cell in the lymph nodes following therapy. The study identified some factors associated with a higher chance of therapy response. This study is the largest of its kind in Romania, and its results are similar to those of other studies on Her2-positive breast cancer.

## 1. Introduction

Breast cancer is the second most commonly diagnosed type of cancer, with an estimated 2.3 million new cases in 2022, being only surpassed by lung cancer. Overall, for both sexes, breast cancer is the fourth leading cause of cancer-associated death. In women, breast cancer is the most commonly diagnosed cancer and the leading cause of cancer-associated mortality [1].

Her2-positive invasive breast cancer (IBC) represents around 15% of IBC cases, and several factors have been found to influence Her2-positivity rates in IBC [2,3,4]. Her2-positive IBC is considered one of the more aggressive breast cancer types. The addition of Her2-targeted therapy (trastuzumab) to standard chemotherapy has changed the natural history of this type of cancer. In early-stage breast cancer, targeted therapy reduces the recurrence risk of IBC and the mortality associated with IBC by a third [5,6].

Pathological complete response (pCR) following neoadjuvant therapy (NAT) for IBC showed a strong correlation with event-free survival and overall survival, with the strongest association observed for triple-negative breast cancer and Her2-positive breast cancer [5,7]. Improvements in pCR rates in Her2-positive IBC have been observed after the introduction of the “dual blockade” (trastuzumab + pertuzumab) [8].

Across Europe, incidence and age-standardized mortality rates have shown high variability, and “real-world” data regarding breast cancer therapy response in Central and Eastern Europe are limited compared to data from Western and Northern Europe [9]. In the last three decades, the five-year net survival from breast cancer has shown a net ascending trend, but several Eastern European countries have lower survival rates [9,10]. Data from Romania, especially for Her2-positive breast cancer response to therapy, are particularly scarce [11,12,13,14,15,16,17,18,19,20].

The aim of our study was to evaluate the response to NAT using chemotherapy and Her2-targeted therapy in a cohort of 167 patients diagnosed with invasive breast cancer in our institution.

## 2. Materials and Methods

For patient selection, we reviewed all records of breast pathology examined in our pathology department between January 2020 and September 2024. In this study, we included female patients diagnosed with invasive breast cancer (IBC) by means of histopathology performed on biopsy specimens at the pathology department of our institution. The study only included patients with Her2-positive IBC defined based on the current American Society of Clinical Oncology/College of American Pathologists (ASCO/CAP) recommendation (tumors with IHC score 3+ or tumors with IHC scores 2+ and ISH Amplified/Positive Groups 1 and 3) [21,22]. Only patients who benefited from neoadjuvant targeted anti-Her2 therapy were selected (either in our institution or in other medical centers (n = 6) with available data regarding the therapeutic regimen). Only those patients who underwent the surgical phase of the treatment in our institution and for whom the resection specimen was examined in the department of pathology were eligible. The study included patients with accessible prognostic and predictive markers, as well as clinical staging data prior to therapy. Metastatic patients were excluded from the study.

The pathological complete response (pCR) assessed on the resection specimen was defined as the absence of invasive carcinoma in the breast or axillary lymph node resected tissue following neoadjuvant therapy [23].

Statistical analysis was performed using Microsoft Excel for Microsoft 365 MSO (version 2504 Build 16.0.18730.20122) and R version 4.1.2 (R Foundation for Statistical Computing, Vienna, Austria). Continuous variables were compared by means of Student’s *t*-test, and categorical variables were compared using the Chi-squared or Fisher’s exact tests.

## 3. Results

This study analyzed 2248 histopathology reports of IBC, which included initial biopsies from primary tumors, axillary lymph node biopsies, and resection specimens. The overall Her2-positivity rate for IBC when comprising all the analyzed specimens was 16.90% (calculated as the percentage of positive specimens (n = 386) from the total number of samples analyzed (n = 2248)).

From these initial data, 167 consecutive female patients diagnosed with Her2-positive invasive breast cancer (IBC) who met the inclusion criteria were selected. Patient age ranged between 29 and 82 years, with an average of 54.36 years and a median of 53 years. Approximately one-third of the patients (31%, n = 52) were diagnosed in the sixth decade of life, and nearly 81% of cases (n = 135) were diagnosed in the interval between the fifth and seventh decades of life.

More than half of the cases were clinical Stage III cases (55%, n = 92 cases). Clinical lymph node involvement was identified in 84% of the cases (n = 140). Approximately two-thirds of the cases exhibited high levels of Her2 protein expression (3+ immunohistochemistry (IHC) score), and the rest showed an equivocal level of protein expression (2+ IHC score), requiring reflex in situ hybridization testing (ISH), with the final classification of all cases considered Her2-positive after combining IHC and ISH analyses. Most cases demonstrated hormone receptor expression (HR) (82%, n = 137). All patients received standard-of-care neoadjuvant therapy represented by anthracycline–taxanes or carboplatin–taxanes-based combination therapy with associated HER2-targeted agents and endocrine therapy according to HR status (Table 1).

**Figure 1:**
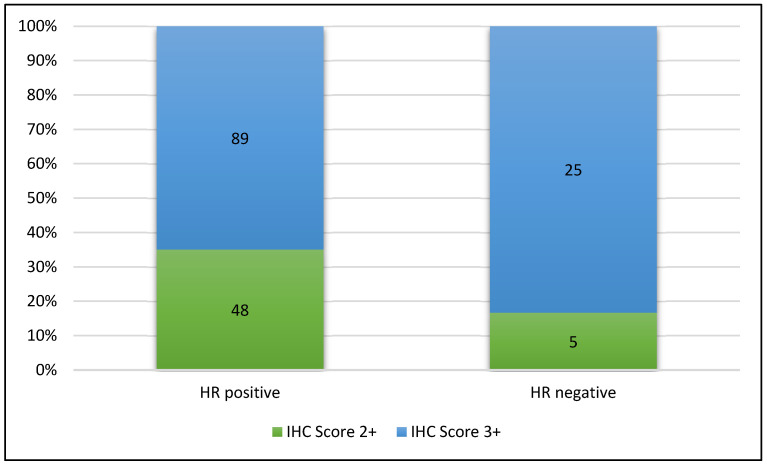
Combined analysis of the Her2 IHC score and HR status showing a higher proportion of HR-negative cases in the 3+ HER2-IHC category.

Hormone receptor (HR) status showed that most cases were HR-positive (82%, n = 137). Combined analysis of the IHC score and HR status, presented in Figure 1, showed a higher frequency of HR-negative (HR (−)) cases in the 3+ Her2-IHC score category versus the lower frequency observed in the 2+ Her2-IHC group (21.93%, n = 25 vs. 9.43%, n = 5). The differences in the frequency of HR (−) cases in the two groups were marginal in terms of statistical significance (*p* = 0.054, Fisher’s exact test, 95% CI [0.95, 9.74]).

Overall, the pCR rate was 50.29% (n = 84), with higher pCR rates observed in the Her2-IHC 3+ cases, HR (−) cases, ER (−) cases, and with marginal significance PR (−) cases (Table 2).

Patient age did not influence pCR in our series (*p* > 0.05, Student’s *t*-test). We found no statistically significant differences in pCR rates between clinical Stage II and Stage III cases (57.33% in Stage II cases vs. 44.57% in Stage III cases, *p* > 0.05, Fisher’s exact test); however, when we filtered for 3+ Her2-IHC cases, we found that Stage II cases had a significantly higher pCR rate (Stage II pCR = 74.51% vs. Stage III pCR = 52.38%, *p* = 0.019). Cases with a 2+ Her2-IHC score showed no stagewise significant differences in response rates (*p* > 0.05, Fisher’s exact test).

Of the 140 cases clinically classified as having lymph node involvement (>cN0), 79.29% were classified as pN0 (n = 111) after pathological examination of the resection specimen. In the 3+ Her-IHC group with clinical lymph node involvement (>cN0, 3+ Her2-IHC, n = 102), most cases were classified as pN0 (86.27%, n = 88) after pathological examination of the resection specimen, and in the 2+ Her-IHC group with clinical lymph node deposits (>cN0, 2+ Her2-IHC n = 38), most cases were classified as pN0 (60.52%, n = 23) after pathological examination, suggesting a higher control of lymph node diseases in 3 + Her2-IHC cases (*p* = 0.0018, Fisher’s exact test).

Ki-67 had a significantly higher value in pCR cases (average Ki-67 = 42.23%) compared to cases with partial response (average Ki-67 = 35.38%) (*p* = 0.008, Student’s *t*-test).

There were no statistically significant differences in pCR rates between cases included in ASCO/CAP ISH Group 1 compared to cases included in the ASCO/CAP ISH Group 3. We compared pCR and pPR cases and found no statistically significant differences when analyzing the average HER2 copy number and the average HER2:CEP17 ratios (ratios between HER2 and centromeric probe copy number for chromosome 17 for individual cases) in the two groups (Student’s *t*-test, *p* > 0.05).

According to multivariable regression analysis of cases with an IHC 3+ score, those with a high Ki-67 value had a higher chance of achieving pCR, while advanced stage and hormone receptor positivity were associated with lower pCR rates (Table 3).

## 4. Discussion

Pathological complete response (pCR) has been used as a surrogate for clinical outcomes in IBC. Patients who achieve pCR after neoadjuvant therapy have a 5-year disease-free survival of 85% [24]. Achieving pCR after systemic therapy has been suggested to have a similar prognostic value regardless of the therapeutic regime used [25]. Patients who achieve pCR following neoadjuvant therapy with high clinical tumor stage (cT3 and cT4) have a higher risk of relapse compared to those at early stages [26].

The overall pCR rate observed in our study was similar to that reported in previously published studies [24,27,28,29]. Luz et al. reported data from four countries, with pCR rates ranging from 55.3% to 68.5%, but with no statistically significant differences between study groups from each country (*p* > 0.05) [24]. Krystel-Whittemore et al. reported a pCR rate of 59% with higher rates in IHC 3+ cases compared to 2+, in Grade 3 tumors, PR-negative tumors, and in patients treated with dual anti-HER2 therapy. The slightly lower value for the pCR rate observed in our group can be explained by several factors, such as high clinical stage, high frequency of lymph node involvement, high number of HR (+) cases, and frequency of 2+ HER2-IHC cases.

Our study also showed a high response in 3+Her2-IHC cases with significantly higher pCR rates compared to 2+ Her2-IHC/ISH positive cases. Higher pCR rates were also associated with HR (–) tumors, lower clinical stage, ER (–) tumors, and high Ki-67 values. A higher pCR rate was observed in PR (−) tumors, with a marginal statistical significance. Several other studies have identified these parameters as having a predictive value for pCR [24,27,30].

Variations in pCR rates can be due to the therapy regime employed, as studies have shown higher pCR rates in patients treated with chemotherapy with added anti-HER2 dual blockade compared to trastuzumab-only regimens [8,28,29,31]. However, Jiao et al. observed a limited effectiveness of neoadjuvant trastuzumab and pertuzumab therapy for 2+ HER2-IHC/ISH positive IBC, compared to 3+ HER2-IHC cases and attributed this finding to subtype heterogeneity [27,32].

Patient age, ISH group, HER2 copy number, and HER2:CEP17 ratio did not seem to influence pCR rates in our study. Previous studies have indicated that ISH Group 1 cases have a higher pCR rate compared to Group 3 cases in IHC equivocal cases (2+ Her-IHC) and in cases with high HER2:CEP17 ratios [33,34,35]. An analysis from the NeoALTTO trial showed that the HER2 copy number is a predictive factor for pCR in unselected cases, but after adjusting for IHC expression, the predictive value of this variable diminishes [36]. Other factors cited as predictors of pCR following NAT include high levels of HER2 mRNA with increased HER2 signaling and immune cell infiltration, while HER2 heterogeneity was associated with the lack of pCR or lower pCR rates [32,37].

Axillary lymph node disease control (axillary complete pathological response, ypN0) was observed in 79% of the cases, with higher values in IHC 3+ cases compared to IHC 2+ cases with ISH amplification. A meta-analysis including 33 studies and 57531 breast cancer cases showed a high axillary pathological response rate of 60% for Her2-positive HR (−) cases, followed by Her2-positive cases with an axillary pathological response rate of 59% [38]. The slightly higher values in our study must be viewed with caution, as most cases were not confirmed by means of lymph node biopsy and histopathological examination.

Studies in the region regarding response to NAT therapy in breast cancer are limited. A study from Thessaloniki, Greece, found a response rate of 67.5% of 77 HER2-positive patients. Higher pCR rates were observed in HR (−) negative cases (80.6%). The study highlighted lobular or mixed lobular and ductal breast cancer and HR positivity to be inversely related to pCR, a similar observation to our study [39]. Another study from Southern Europe, in Italy, Neopearl, involved a nationwide collaboration recruiting 271 patients with HER-2 positive Stage II-III breast cancer, with a pCR rate of 55.7%. The study included patients treated with trastuzumab and chemotherapy and dual-blockade and chemotherapy cohorts, and in univariate analysis, they found that negative ER and PgR status, HER2 3+ score at IHC, and treatment with pertuzumab were significantly associated with an increased pCR rate. In multivariate analysis, only HER2 expression and pertuzumab therapy retained the predictive value [40]. Similar findings were revealed in our study, but as the number of cases treated with trastuzumab and chemotherapy was very small, a comparison between the two types of targeted therapy was not possible. A study from Hungary involving 82 Stage II and III HER2+ early breast cancer patients treated with NAT, which included dual-blockade pertuzumab and trastuzumab, showed a pCR of 54%. Ki-67 values and the neutrophil–lymphocyte ratio were identified as predictors for pCR [41]. A study from Turkey reported a pCR rate of 51,42% for axillary nodes and primary breast tumor and identified the neutrophil–lymphocyte ratio, the platelet–lymphocyte ratio, prognostic nutritional index ratio, dual-blockade therapy and HR-negative status as significant predictors of pCR [42].

According to a report from 2023 regarding the cancer burden in Romania by the National Institute of Public Health and the National Center for Non-Communicable Disease Surveillance, breast cancer is the most diagnosed form of cancer in women, in Romania, and is the third leading cause of cancer-related mortality. Although the amount of funding for cancer has increased in recent years, the total per capita cost of cancer in Romania (PPP adjusted) up until 2023 was about half of the EU-27 average. Over the 2011–2020 decade, cancer mortality dropped by 1.8%, at a much slower rate than the 9.4% EU-27 average. In the same decade, six of the most common ten cancers registered an increase in cancer-related death rates, including breast cancer. The number of disability-adjusted life years (DALYs) lost due to cancer in Romania is 46% higher than the EU-27 average. While the gap narrowed from 70% in 2000 to 46% in 2019, the reduction was more significant among men compared to women. As of 2023, the oncology drug list in Romania contained 162 approved molecules. Although the list of new molecules was constantly updated for the years 2020–2023, the time from central approval to drug availability for Romania was on average 828 days, compared to an average of 531 days across the EU. Concerns are growing regarding the financial sustainability of the health system, which is also threatened by the increasing number of cancer patients and the costs of new oncology drugs [43,44].

The present study has several limitations. First, the limited number of cases, especially for the 2+ HER2-IHC category, had an impact on the comparability of several groups. The limited number of cases has to also be considered in light of the fact that about half the study period was during the COVID-19 pandemic, which meant that, at least for a period of time, there were substantial limitations to patients mobility, with the possibility that some of the patients initially diagnosed with breast cancer at our institution were forced to continue their NAT, at least in part, at an external institution or to perform the surgical phase of the therapy at another institution. The inclusion criteria did not allow us to include such patients in the current study. Another important limitation of our study is that it does not provide information regarding adjuvant therapy, clinical follow-up, or survival.

Our study uses pCR as a measure to assess response to therapy, a surrogate for overall and event-free survival. Furthermore, the study is limited to NAT therapy response and does not provide information regarding adjuvant therapy and clinical follow-up. The data presented here are the experience of a single institution, thus limiting their generalization.

The benefits of this single-center study include uniformity regarding breast cancer diagnostics, Her2 testing, and patient therapy. Her2 testing was performed in our institution using automated IHC and Ventana automated stainers, and the 4B5 clone and reflex testing cases were performed using ISH for all 2+ cases. Both IHC and ISH protocols have been published in previous reports [45]. All patients diagnosed with breast cancer were evaluated by a multidisciplinary committee that provides treatment recommendations. Our institution participates in national and international external quality control and accreditation processes.

## 5. Conclusions

The data presented in our study, to the best of our knowledge, encompass the largest cohort of patients diagnosed with HER2-positive IBC from Romania. The presented results and the pCR predictive factors were comparable to those cited in other studies on Her2-positive IBC cases.

## Figures and Tables

**Table 1:** Patient baseline characteristics.

**Clinical Stage**	**Stage II**		**Stage III**		
	45% (n = 75)		55% (n = 92)		
	IIA	IIB	IIIA	IIIB	IIIC
	21% (n = 35)	24% (n = 40)	29.3% (n = 49)	23.3% (n = 39)	2.3% (n = 4)
**Lymph node** **category (N)**	**N0**	**N1**		**N2**	
	18% (n = 30)	49% (n = 82)		33% (n = 55)	
**Hormone** **receptor** **expression**	**HR positive**		**HR negative**		
	82% (n = 137)		18% (n = 30)		
**Her2 IHC** **expression**	**3+**		**2+**		
	68% (n = 114)		32% (n = 53)		
**Her2 ISH Group**	**Group 1**		**Group 3**		
	75% (n = 39)		25% (n = 13)		
**Neoadjuvant therapy**	**Neoadjuvant chemotherapy +** **HER2 Dual Blockade** **(Pertuzumab + Trastuzumab)**		**Neoadjuvant chemotherapy + Trastuzumab**		
	92% (n = 153)		8% (n = 14)		

**Table 2:** Summary of the most significant characteristics influencing pCR rates.

Characteristic	Category	pCR Rates (%)	pCR Cases Count (n)	Statistical Significance (Fisher’s Exact Test *p* Value)
**Overall**	-	50.29	114	-
**Her2 protein** **expression**	3+ Her2-IHC	62.28	71	<0.001 95% CI [0.09, 0.43]
	2+ Her2-IHC	24.53	13	
**Hormone** **receptor** **expression**	HR (+)	45.25	62	0.008 95% CI [0.10, 0.76]
	HR (−)	73.33	22	
**Estrogen** **receptor** **expression**	ER (+)	10.84	9	0.001 95% CI [0.10, 0.62]
	ER (−)	32.14	27	
**Progesterone** **receptor** **expression**	PR (+)	33.73	28	0.059 95% CI [0.27, 1.04]
	PR (−)	32.14	27	

**Table 3:** Logistic regression analysis for pathological complete response.

Variable	Estimate	OR	95% CI	*p* Value
Age category (>50)	0.057	1.06	[0.52–2.13]	0.875
Clinical stage (Stage III)	−0.712	0.49	[0.24–0.99]	0.046
HR_status (HR(+)cases)	−1.101	0.33	[0.13–0.86]	0.023
Ki-67 (>25%)	1.122	3.07	[1.41–6.66]	0.005
IHC Score (3+ cases)	1.690	5.42	[2.45–11.99]	<0.001

## Data Availability

The data presented in this study are available upon request from the corresponding author.

## Abbreviations

The following abbreviations are used in this manuscript:

pCR	Pathological complete response
IBC	Invasive breast cancer
Her2	Human epidermal growth factor receptor 2
CEP17	Centromeric enumeration probe of chromosome 17
NAT	Neoadjuvant therapy
ISH	In situ hybridization
IHC	Immunohistochemistry
EU	European Union
ASCO/CAP	American Society of Clinical Oncology/College of American Pathologists

## References

[1] Bray F., Laversanne M., Sung H., Ferlay J., Siegel R.L., Soerjomataram I., Jemal A. (2024). Global cancer statistics 2022: GLOBOCAN estimates of incidence and mortality worldwide for 36 cancers in 185 countries. CA A Cancer J. Clin..

[2] Rüschoff J., Lebeau A., Kreipe H., Sinn P., Gerharz C.D., Koch W., Morris S., Ammann J., Untch M. (2017). Assessing HER2 testing quality in breast cancer: Variables that influence HER2 positivity rate from a large, multicenter, observational study in Germany. Mod. Pathol..

[3] Lin C.Y., Carneal E.E., Lichtensztajn D.Y., Gomez S.L., Clarke C.A., Jensen K.C., Kurian A.W., Allison K.H. (2017). Regional Variability in Percentage of Breast Cancers Reported as Positive for HER2 in California: Implications of Patient Demographics on Laboratory Benchmarks. Am. J. Clin. Pathol..

[4] Kim M.C., Cho E.Y., Park S.Y., Lee H.J., Lee J.S., Kim J.Y., Lee H.C., Yoo J.Y., Kim H.S., Kim B. (2024). A Nationwide Study on HER2-Low Breast Cancer in South Korea: Its Incidence of 2022 Real World Data and the Importance of Immunohistochemical Staining Protocols. Cancer Res. Treat. Off. J. Korean Cancer Assoc..

[5] Loibl S., André F., Bachelot T., Barrios C., Bergh J., Burstein H., Cardoso M., Carey L., Dawood S., Del Mastro L. (2024). Early breast cancer: ESMO Clinical Practice Guideline for diagnosis, treatment and follow-up. Ann. Oncol..

[6] (2021). Trastuzumab for early-stage, HER2-positive breast cancer: A meta-analysis of 13 864 women in seven randomised trials. Lancet Oncol..

[7] Cortazar P., Zhang L., Untch M., Mehta K., Costantino J.P., Wolmark N., Bonnefoi H., Cameron D., Gianni L., Valagussa P. (2014). Pathological complete response and long-term clinical benefit in breast cancer: The CTNeoBC pooled analysis. Lancet.

[8] Gianni L., Pienkowski T., Im Y.H., Roman L., Tseng L.M., Liu M.C., Lluch A., Staroslawska E., de la Haba-Rodriguez J., Im S.A. (2012). Efficacy and safety of neoadjuvant pertuzumab and trastuzumab in women with locally advanced, inflammatory, or early HER2-positive breast cancer (NeoSphere): A randomised multicentre, open-label, phase 2 trial. Lancet Oncol..

[9] Allemani C., Matsuda T., Di Carlo V., Harewood R., Matz M., Nikšić M., Bonaventure A., Valkov M., Johnson C.J., Estève J. (2018). Global surveillance of trends in cancer survival 2000–14 (CONCORD-3): Analysis of individual records for 37 513 025 patients diagnosed with one of 18 cancers from 322 population-based registries in 71 countries. Lancet.

[10] Elmadani M., Mokaya P.O., Omer A.A., Kiptulon E.K., Klara S., Orsolya M. (2025). Cancer burden in Europe: A systematic analysis of the GLOBOCAN database (2022). BMC Cancer.

[11] Pantelimon I., Stancu A.M., Coniac S., Ionescu A.I., Atasiei D.I., Georgescu D.E., Galeș L.N. (2025). Local Control of Advanced Breast Cancer-Debate in Multidisciplinary Tumor Board. J. Clin. Med..

[12] Ionescu Miron A.I., Anghel A.V., Antone-Iordache I.L., Atasiei D.I., Anghel C.A., Barnonschi A.A., Bobolocu A.M., Verga C., Șandru F., Lișcu H.D. (2024). Assessing the Impact of Organ Failure and Metastases on Quality of Life in Breast Cancer Patients: A Prospective Study Based on Utilizing EORTC QLQ-C30 and EORTC QLQ-BR45 Questionnaires in Romania. J. Pers. Med..

[13] Miron A.-I., Anghel A.-V., Barnonschi A.-A., Mitre R., Liscu H.-D., Găinariu E., Pătru R., Coniac S. (2023). Real-world outcomes of CDK4/6 inhibitors treatment in metastatic breast cancer in Romania. Diagnostics.

[14] Coca R., Moisin A., Coca R., Diter A., Racheriu M., Tanasescu D., Popa C., Cerghedean-Florea M.E., Boicean A., Tanasescu C. (2024). Exploring Therapeutic Challenges in Patients with HER2-Positive Breast Cancer-A Single-Center Experience. Life.

[15] Simionescu A.A., Horobeț A., Belaşcu L., Median D.M. (2020). Real-world data analysis of pregnancy-associated breast cancer at a tertiary-level hospital In Romania. Medicina.

[16] Lungulescu C.V., Camen G.-C., Naidin M.-S., Berisha T.-C., Bita A., Dinescu V.-C., Buteica S.A., Dimulescu M.-D., Volovat S.R., Turcu-Stiolica A. (2024). Real-World Efficacy and Adherence to Palbociclib in HR-Positive, HER2-Negative Advanced Breast Cancer: Insights from a Romanian Cohort. Cancers.

[17] Oprean C.M., Badau L.M., Petrita R., Median M.D., Dema A. (2025). Real-World, National Study of Palbociclib in HR+/HER2− Metastatic Breast Cancer: A 2.5-Year Follow-Up PALBO01/2021. Diagnostics.

[18] Cătană A., Trifa A.P., Achimas-Cadariu P.A., Bolba-Morar G., Lisencu C., Kutasi E., Chelaru V.F., Muntean M., Martin D.L., Antone N.Z. (2023). Hereditary Breast Cancer in Romania—Molecular Particularities and Genetic Counseling Challenges in an Eastern European Country. Biomedicines.

[19] Pop L.-A., Cojocneanu-Petric R.-M., Pileczki V., Morar-Bolba G., Irimie A., Lazar V., Lombardo C., Paradiso A., Berindan-Neagoe I. (2018). Genetic alterations in sporadic triple negative breast cancer. Breast.

[20] Mustata L.M., Peltecu G., Mugescu D.C., Nedelea F.M., Median M.D. (2024). Single Center Experience of Genetic Testing in Patients Undergoing Breast Cancer Treatment. Maedica.

[21] Wolff A.C., Hammond M.E.H., Allison K.H., Harvey B.E., Mangu P.B., Bartlett J.M.S., Bilous M., Ellis I.O., Fitzgibbons P., Hanna W. (2018). Human Epidermal Growth Factor Receptor 2 Testing in Breast Cancer: American Society of Clinical Oncology/College of American Pathologists Clinical Practice Guideline Focused Update. J. Clin. Oncol..

[22] Wolff A.C., Somerfield M.R., Dowsett M., Hammond M.E.H., Hayes D.F., McShane L.M., Saphner T.J., Spears P.A., Allison K.H. (2023). Human Epidermal Growth Factor Receptor 2 Testing in Breast Cancer. Arch. Pathol. Lab. Med..

[23] Sahoo S., Lester S.C. (2009). Pathology of breast carcinomas after neoadjuvant chemotherapy: An overview with recommendations on specimen processing and reporting. Arch. Pathol. Lab. Med..

[24] Luz P., Lopes-Brás R., de Pinho I.S., Patel V., Esperança-Martins M., Gonçalves L., Gonçalves J., Freitas R., Simão D., Galnares M.R. (2025). Predictive factors for pCR and relapse following neoadjuvant dual HER2-blockade in HER2+ breast cancer: An international cohort study. Clin. Transl. Oncol..

[25] Nierenberg T.C., Thomas S.M., Halliday I., Botty van den Bruele A., Chiba A., Modell Parrish K.J., Woriax H.E., DiNome M.L., Westbrook K.E., Plichta J.K. (2025). Survival outcomes after pathologic complete response with neoadjuvant endocrine therapy vs. neoadjuvant chemotherapy: A retrospective national database study. Breast Cancer Res. Treat..

[26] Huober J., van Mackelenbergh M., Schneeweiss A., Seither F., Blohmer J.U., Denkert C., Tesch H., Hanusch C., Salat C., Rhiem K. (2023). Identifying breast cancer patients at risk of relapse despite pathological complete response after neoadjuvant therapy. NPJ Breast Cancer.

[27] Krystel-Whittemore M., Xu J., Brogi E., Ventura K., Patil S., Ross D.S., Dang C., Robson M., Norton L., Morrow M. (2019). Pathologic complete response rate according to HER2 detection methods in HER2-positive breast cancer treated with neoadjuvant systemic therapy. Breast Cancer Res. Treat..

[28] Hurvitz S.A., Martin M., Symmans W.F., Jung K.H., Huang C.S., Thompson A.M., Harbeck N., Valero V., Stroyakovskiy D., Wildiers H. (2018). Neoadjuvant trastuzumab, pertuzumab, and chemotherapy versus trastuzumab emtansine plus pertuzumab in patients with HER2-positive breast cancer (KRISTINE): A randomised, open-label, multicentre, phase 3 trial. Lancet Oncol..

[29] Rodríguez M., González D.M., El-Sharkawy F., Castaño M., Madrid J. (2023). Complete pathological response in patients with HER2 positive breast cancer treated with neoadjuvant therapy in Colombia. Biomedica.

[30] Hännikäinen E.-N., Mattson J., Karihtala P. (2023). Predictors of successful neoadjuvant treatment in HER2-positive breast cancer. Oncol. Lett..

[31] Gianni L., Pienkowski T., Im Y.H., Tseng L.M., Liu M.C., Lluch A., Starosławska E., de la Haba-Rodriguez J., Im S.A., Pedrini J.L. (2016). 5-year analysis of neoadjuvant pertuzumab and trastuzumab in patients with locally advanced, inflammatory, or early-stage HER2-positive breast cancer (NeoSphere): A multicentre, open-label, phase 2 randomised trial. Lancet Oncol..

[32] Jiao D., Li G., Dai H., Wang J., Zhang J., Hou Y., Guo X., Zhao Y., Gong X., Liu Z. (2024). Comparison of the response to neoadjuvant therapy between immunohistochemistry HER2 (3+) and HER2 (2+)/ISH+ early-stage breast cancer: A retrospective multicenter cohort study. Oncologist.

[33] Antolín S., García-Caballero L., Reboredo C., Molina A., Mosquera J., Vázquez-Boquete Á., Gallego R., Santiago M.P., Concha Á., Pérez E. (2021). Is there a correlation between HER2 gene amplification level and response to neoadjuvant treatment with trastuzumab and chemotherapy in HER2-positive breast cancer?. Virchows Arch..

[34] Choi J.H., Jeon C.W., Kim Y.O., Jung S. (2020). Pathological complete response to neoadjuvant trastuzumab and pertuzumab therapy is related to human epidermal growth factor receptor 2 (HER2) amplification level in HER2-amplified breast cancer. Medicine.

[35] Li F., Ju Q., Gao C., Li J., Wang X., Yan M., Zhang L., Huang M., Long Q., Jin X. (2022). Association of HER-2/CEP17 ratio and HER-2 copy number with pCR rate in HER-2-positive breast cancer after dual-target neoadjuvant therapy with trastuzumab and pertuzumab. Front. Oncol..

[36] Venet D., Rediti M., Maetens M., Fumagalli D., Brown D.N., Majjaj S., Salgado R., Pusztai L., Harbeck N., El-Abed S. (2021). Copy number aberration analysis to predict response to neoadjuvant Anti-HER2 therapy: Results from the NeoALTTO Phase III clinical trial. Clin. Cancer Res..

[37] Li Z., Metzger Filho O., Viale G., dell’Orto P., Russo L., Goyette M.-A., Kamat A., Yardley D.A., Abramson V.G., Arteaga C.L. (2024). HER2 heterogeneity and treatment response–associated profiles in HER2-positive breast cancer in the NCT02326974 clinical trial. J. Clin. Investig..

[38] Samiei S., Simons J.M., Engelen S.M.E., Beets-Tan R.G.H., Classe J.M., Smidt M.L. (2021). Axillary Pathologic Complete Response After Neoadjuvant Systemic Therapy by Breast Cancer Subtype in Patients With Initially Clinically Node-Positive Disease: A Systematic Review and Meta-analysis. JAMA Surg..

[39] Papazisis K.T., Liappis T., Kontovinis L., Pouptsis A., Intzes S., Natsiopoulos I. (2020). Real-world experience of neoadjuvant chemotherapy for early breast cancer patients: An observational non-interventional study in Thessaloniki, Greece. J. BUON.

[40] Fabbri A., Nelli F., Botticelli A., Giannarelli D., Marrucci E., Fiore C., Virtuoso A., Scagnoli S., Pisegna S., Alesini D. (2023). Pathlogic response and survival after neoadjuvant hemotherapy with or without pertuzumab in patients with HER2-Positive breast cancer: The neopearl nationwide collaborative study. Front. Oncol..

[41] Boér K., Kahán Z., Landherr L., Csőszi T., Máhr K., Ruzsa Á., Horváth Z., Budai B., Rubovszky G. (2021). Pathologic complete response rates after neoadjuvant pertuzumab and trastuzumab with chemotherapy in early stage HER2-positive breast cancer- increasing rates of breast conserving surgery: A real-world experience. Pathol. Oncol. Res..

[42] Gulmez A., Harputluoglu H. (2022). Effect of Inflammatory Markers on the Pathologic Complete Response in the Neoadjuvant Treament of HER-2 Positive Local Advanced Breast Cancer. Eurasian J. Med. Investig..

[43] IQVIA, EFPIA (2025). EFPIA Patients W.A.I.T. Indicator 2024 Survey. https://efpia.eu/media/oeganukm/efpia-patients-wait-indicator-2024-final-110425.pdf.

[44] National Institute of Public Health (2023). Cancer Country Profile—Romania 2023. https://insp.gov.ro/wp-content/uploads/2024/03/Profil-de-tara-privind-cancerul-2023.pdf.

[45] Fetica B., Blaga M.L., Trifa A.P., Bocean C.M., Balacescu O., Fulop A., Pop B. (2025). FICTION Technique—A Candidate for the Assessment of HER2 Status in Breast Invasive Carcinomas. Medicina.

